# Illness perceptions predict mortality in patients with predialysis chronic kidney disease: a prospective observational study

**DOI:** 10.1186/s12882-020-02189-7

**Published:** 2020-12-10

**Authors:** Priscilla Muscat, John Weinman, Emanuel Farrugia, Liberato Camilleri, Joseph Chilcot

**Affiliations:** 1 Renal Unit, Nephrology Department, Mater Dei Hospital, Msida, MSD 2090 Malta; 2 Health Psychology Section, Psychology Department, Institute of Psychiatry, Psychology and Neuroscience, King’s College London, 5th Floor Bermondsey Wing, Guy’s Campus, London Bridge, London, SE1 9RT UK; 3 Institute of Pharmaceutical Science, Pharmaceutical Sciences Clinical Academic Group King’s College London, 5th floor, Franklin -Wilkins Building. 150 Stamford Street, London, SE19NH UK; 4 Statistics and Operations Research Department University of Malta, Msida, MSD 2080 Malta

**Keywords:** Illness perception, Chronic kidney disease, CKD, Predialysis, Depression, Distress, Mortality

## Abstract

**Background:**

Illness perceptions have been shown to predict a range of psychosocial and clinical outcomes in kidney disease; including quality of life, distress, treatment adherence and even survival in end-stage renal disease patients on dialysis. The aim of this study was to evaluate whether illness perceptions impact mortality in incident predialysis Chronic Kidney Disease (CKD) patients.

**Methods:**

Over the study period between September 2015 and June 2019, a total of 200 participants with predialysis CKD were recruited from the Nephrology Outpatient’s clinics at Mater Dei Hospital, Malta. The participants were followed up until June 2019, and the mortality information was collected. Cox proportional hazards models were used to examine the association between illness perceptions, and mortality risk, after adjustment for covariates including distress, kidney function, co-morbidity and psychological distress.

**Results:**

Of the 200 cases available for analysis, there were 43 deaths. The mean survival time was 718.55 days (min. 3 days, max. 1297 days). The cumulative survival 1-year post the assessment of the Revised Illness Perceptions Questionnaire (IPQ–R) was 93%. Stronger identity beliefs (HR = 1.199, 95% CI: 1.060–1.357, *p* = 0.004), perceptions of a chronic timeline (HR = 1.065, 95% CI: 1.003–1.132, *p* = 0.041), personal control beliefs (HR = 0.845, 95% CI: 0.748–0.955, *p* = 0.007) and perceptions of control over the treatment (HR = 0.812, 95% CI: 0.725–0.909, *p* = 0.000) demonstrated a significant association with mortality after controlling covariates.

In a subsequent saturated model, perceived identity, chronic timeline and treatment control perceptions remained significant predictors of mortality, together with serum albumin, comorbidities and urea.

**Conclusions:**

CKD patients’ perceptions of treatment control, perceptions of a chronic timeline and perceived illness identity predict survival independently of clinical prognostic factors, including kidney function and co-morbidity. Illness perceptions are important and potentially modifiable risk factors in CKD. Further studies are required to test whether the assessment and the implementation of psychological interventions aimed to modify maladaptive illness perceptions influence clinical outcomes in CKD.

## Background

Chronic Kidney Disease (CKD) is a worldwide health problem which is found to affect around 14.5% of the global population and is associated with increased morbidity risk and mortality rates [1, 2]. Literature looking into life expectancy and mortality in predialysis CKD, has tended to focus mostly on the role of clinical factors within this cohort. The data by Turin et al., [3] revealed that the life expectancy in predialysis CKD is reduced significantly for all levels of renal function below an eGFR of 60 ml/min/1.73 m^2^, with the most notable decline in life expectancy resulting in those patients with eGFR< 30 ml/min/1.73 m^2^. Extra renal comorbidities have also been associated with morbidity and mortality in CKD [4].

Besides the clinical factors, studies have also explored the role of psychosocial factors on mortality; whereby psychological factors have been identified as predictors of short-term survival in End Stage Kidney Disease (ESKD) through their influence on self-management behaviours, which are essential for effective and holistic management of Kidney Disease and its treatment. Illness perceptions and depression have been associated with survival and mortality in ESKD patients receiving dialysis [5, 6]. The predictive role of illness perceptions has also been confirmed in multiple patient groups, including patients with cardiovascular disease [7, 8], diabetes mellitus [9], gout [10] and cancer [11].

Whilst this association has been established in other medical cohorts and in patients with ESKD on dialysis, more literature is needed on earlier stages of CKD to confirm whether this association is also present and if so, psychological care needs to be integrated as part of the predialysis CKD routine care.

When individuals develop a physical disease, they tend to generate a specific pattern of beliefs, representations or perceptions of their illness. These mental representations are conceptualised within a framework of self-regulation known as the Common-Sense Self-Regulation Model (CS-SRM) [12, 13]. According to the model, people make sense of a health threat by developing their own cognitive and emotional representations also known as illness beliefs or illness perceptions of a health threat. The illness perceptions are thus processed in three stages namely; interpretation, whereby the individual forms illness perceptions based on intrinsic factors such as demographics, knowledge or education and symptom perception. This is followed by coping procedures, which enables the individual to identify strategies to reduce the health threat associated with the disease and finally appraisal takes place whereby the individual then analyses the outcomes of the adopted coping strategies [14].

Research exploring the association between illness perceptions and mortality in ESKD has confirmed that patients’ beliefs are associated with mortality. In a study by Kimmel et al., [15], an increase in the number of perceived consequences attributed to the kidney disease was associated with an increased mortality risk in dialysis patients. Poor treatment control was also significantly associated with mortality in end-stage kidney disease [5, 16]. In contrast, Peterson et al., [17] found no significant difference in illness perceptions upon comparing a sample of ESKD survivors and non survivors.

Depression has also been associated with a variety of poor outcomes in ESKD, including increased hospitalisation, poor quality of life, and increased morbidity amongst predialysis CKD patients, after controlling for demographic and clinical covariates [18]. Similarly, depressive symptoms have been shown to predict mortality in End-Stage Kidney Disease cohorts on dialysis [5, 6]. Proposed causal mechanisms to explain these poor outcomes in relation to depression include inflammation, as well as non-adherence to therapy, an unhealthy lifestyle and poor nutrition [5, 6, 19].

In summary, illness perceptions and depression have been identified as predictors of short-term survival in ESKD on dialysis, yet few studies have explored these associations in CKD patients. Research evidence from other clinical populations suggests that illness perceptions are modifiable through psychological interventions [20]. The aim of this study was to evaluate whether illness perceptions impact mortality in incident predialysis CKD patients on controlling for covariates.

## Method

### Study population and data sources

The study enrolled patients with CKD treated by Nephrologists at the Mater Dei Hospital, the main general hospital in Malta, a small island with a population of 441,543 people based on the Worldometer [21]. The hospital offers specialized nephrology services, covering the entire medical network area of Malta and Gozo. The study cohort was formed of adult patients over 18 years of age diagnosed with Chronic Kidney Disease and attending clinics from January 2014 to September 2015. A total of 250 participants were approached by the medical team to take part in this study via phone calls or during outpatient clinics. The sample was chosen randomly by the Consultant Nephrologists from the hospital data base of nephrology clinics. Of these, 220 provided informed consent to take part in the study of which 11 participants dropped from the study, 6 participants were ineligible to take part in the study as they had already commenced dialysis treatment and 3 were ineligible due to the presence of cognitive impairment as evidenced by the Mini Mental State Examination, MMSE [22].

### Study cohort

The Inclusion Criteria for the study included age 18 years and over, and a diagnosis of Chronic Kidney Disease at stage 1 to 5 and the exclusion criteria includes patients who have already commenced dialysis, patients that have already been transplanted and severe cognitive impairment. A standardized cognitive assessment was conducted using the Mini Mental State Examination, (MMSE), and a score of < 23 is indicative of a level of cognitive impairment, in which case patients will be excluded.

### Outcome definition

Over the study period (1st September 2015– 30th June 2019), 200 patients diagnosed with Chronic Kidney Disease were followed up with all-cause mortality recorded as the end point. Time-zero in the survival models, was taken at the time patients completed the questionnaire assessments, following recruitment in the study whilst the end point cut off score for all-cause mortality was taken on the 30th June 2019 for all the participants. In the event that the participants passed away in between this time interval, the exact date was extracted from the hospital data system. Therefore, the survival time was assessed from time zero till the date of event (all-cause mortality) or till the end point of the study with the cut off being the 30th June 2019 for all remaining participants.

### Variables

The participants were assessed for their Illness Perceptions of Chronic Kidney Disease at the start of the study upon recruitment.

### Covariates

The following demographic details were also taken including age, gender, ethnicity, time since diagnosis indicated by a categorical variable composed of eight categories (ranging from less or equal to six months, more than six months to one year, around 2 years, around 3 years, around 4 years, around 5 years, five to ten years and more than ten years),treatment or nature of disease management indicated by a categorical variable composed of five categories (ranging from dietary management, 1–2 medications, 3–4 medications, 5–9 medications and 10 or more medications), and level of education. The following clinical data was included in the study; eGFR (calculated using the MDRD equation [23], Serum Creatinine (umol/l), Urea (mmol/l), Serum Albumin (g/l), Haemoglobin (g/dL), C Reactive Protein (CRP) (mg/L) and other extra renal comorbidity was assessed using the Charlson comorbidity index [24]. This information was accessed from their routine blood tests as ordered by their Consultant Nephrologists during their outpatient follow ups. The participants were also assessed for their level of depression and anxiety. All these measures were collected at the start of the study upon recruitment.

### Illness perception measurement

The Revised Illness Perceptions Questionnaire (IPQ–R) [25] was used to assess illness representations of CKD. It has been validated for a range of chronic conditions including renal patients [26]. Illness identity was measured by 14 symptoms in which patients had to report whether they experienced the symptoms and, if so, whether they attributed the symptom to their ‘kidney problem’. “The number of symptoms attributed to their kidney problem were summed (range: 0–14), with higher scores indicating increased illness identity” [25]. “The following dimensions were measured on a 5-point Likert scale (strongly disagree to strongly agree): consequences (e.g. ‘my kidney problem has major consequences on my life’), emotional representations (e.g. ‘I get depressed when I think about my kidney problem’), timeline (e.g. ‘my kidney problem will last for a long time’), cyclical timeline (e.g. ‘my symptoms come and go in cycles’), illness coherence (e.g. ‘my kidney problem is a mystery to me’), personal control (e.g. ‘I have the power to influence my kidney problem’) and treatment control (e.g. ‘my treatment can control my kidney problem’)” [25]. High scores on the timeline and consequence dimensions reflect strongly held beliefs about the chronicity and negative consequences of the condition and the cyclical dimensions reflect strongly held beliefs that the condition is cyclical in nature. High scores on the personal control, treatment control and coherence subscales reflect strong perceptions of illness controllability and a greater personal understanding of the condition. The Cronbach’s alphas are presented in the results section.

### Psychological distress

Given the high correlation between Patient Health Questionnaire (PHQ-9) [27] and the Generalized Anxiety Disorder 7-item scale (GAD-7) [28] a total distress score, termed the PHQADS [29] was used for this study. The PHQ-ADS scale assesses different levels of distress. The following cut off scores will be used; participants with a score of 0–9 to outline minimal levels of distress, 10–19 to outline mild levels of distress, 20–29 to outline moderate distress and 30–48 to outline severe distress. This measure has been validated in a renal sample [6] which showed that the PHQ-ADS is sufficiently unidimensional to warrant a total distress score.

### Ethical considerations

Ethical approval was obtained from the Mater Dei Hospital ethics board, the Psychiatry, Nursing and Midwifery Research Ethics Subcommittee at King’s College London and the University of Malta Research Ethics Committee.

### Survival analysis

All-cause mortality was the outcome measure over the study period (1st September 2015– 30th June 2019). Time-zero in the survival models was taken at the time patients completed the questionnaire assessments. Patients were censored if they regained residual renal function, received a transplant or commenced dialysis treatment, quit the study and if they were still alive by the end of the follow up.

Cox proportional hazards models were constructed to examine the association between illness perceptions and survival. All variables collected including demographic, clinical and psychological factors were evaluated in terms of their association with survival using Cox proportional hazards models.

Adjusted survival models controlled for covariates that demonstrated a significant univariate association with mortality, as evaluated in Cox survival models. The covariates were serum albumin, comorbidity score, urea, and distress scores. The proportion-hazards assumption was tested by entering interaction terms into the survival models, which revealed that the assumption was met. All data were analysed in SPSS version 26.0.

## Results

### Participant demographic and clinical characteristics

A summary of demographic and clinical characteristics is shown in Table 1 (*n* = 200). The mean age of the sample was 69.1 years (+ 13.4). The majority of the sample was males 62.5%. 48.5% of the sample had an eGFR between 30 and 59. The mean comorbidity score was 4.9 (+ 3.1). The level of distress was relatively high with a mean of 18.0 (+ 10.9).

**Table 1:** Demographic and clinical characteristics of participants

Variable	Statistic
***Demographic Characteristics***	
Males, n (%)	125 (62.5)
Females, n (%)	75 (37.5)
Education level:	
Primary n (%)	93 (46.5)
Secondary n (%)	80 (40.0)
Tertiary n (%)	27 (13.5)
Time since Diagnosis	
Less than or equal to 6 months	20 (10.6)
More than 6 months or equal to 1 year	20 (10.6)
Around 2 to 5 years	71 (37.8)
Around 6 to 10 years	31 (16.5)
More than 10 years	46 (24.5)
***Clinical Characteristics*** eGFR n (%)	
60–90	16 (8.0)
30–59	97 (48.5)
15–29	53 (26.5)
10–14	34 (17.0)
Mean Urea ± SD, mmol/l	17.1 + 8.7
Mean Haemoglobin ± SD, g/dl	12.5 + 1.8
Mean Serum Albumin ± SD, g/l	42.9 + 4.4
Mean Extra renal comorbidity ± SD	4.9 + 3.1
Mean Distress Score ± SD	18.0 + 10.9

Table 2 outlines the Cronbach’s alpha for the illness perception items as assessed by the Revised Illness Perceptions Questionnaire (IPQ–R). It also outlines the mean scores and standard deviation for all items and provides an overview of the mean scores for censored participants versus failure that is those who passed away throughout the study period. The participants who passed away had higher observed mean scores for identity, timeline both chronic and cyclical and had also higher mean scores for the perceived consequences and emotional representations. In contrast the participants who passed away during the study had lower mean scores for personal control, treatment control and illness coherence.

**Table 2:** Summary of illness perception scores

					Censored (n-157)		Failure (***n*** = 43)	
Perception	Cronbach’s α	Possible Range	Mean	SD	Mean	SD	Mean	SD
1. Identity	N/A	0–14	3.74	3.28	3.29	2.85	5.37	4.15
2. Timeline- Acute/Chronic	0.95	6–30	22.25	6.21	21.75	6.16	24.07	6.08
3. Timeline -Cyclical	0.92	4–20	9.08	3.33	8.71	3.18	10.44	3.55
4. Consequences	0.91	6–30	17.87	5.70	17.20	5.42	20.33	6.06
5. Treatment Control	0.82	5–25	16.05	3.87	16.76	3.32	13.49	4.61
6. Personal Control	0.84	6–30	18.55	4.56	19.29	4.07	15.84	5.26
7. Illness Coherence	0.79	5–25	16.98	3.66	17.24	3.60	16.02	3.76
8. Emotional Representations	0.94	6–30	19.90	6.27	19.08	6.22	22.88	5.58

### Survival analysis

Patients were followed up for a mean of 718.55 days (min. 3 days, max. 1297 days) during which there were 43 deaths (21.5%) over the whole study period. The cumulative survival 1-year post-IPQ-R assessment was 93%. Over the study period, 3 patients were transferred to other renal units (lost to follow-up), 2 regained residual renal function and 8 were transplanted.

### Unadjusted survival analysis

Table 3 outlines the univariate analysis of demographic and clinical kidney data with all-cause mortality. In the unadjusted Cox proportional hazards model, albumin (HR = 0.874, 95% CI: 0.822–0.928, *p* = 0.000), comorbidities (HR = 1.277, 95% CI: 1.179–1.384), *p* = 0.000), urea, (HR = 1.022, 95% CI: 1.009–1.035, *p* = 0.001) and distress score (HR = 1.087, 95% CI: 1.048–1.128, *p* = 0.000) were significantly associated with mortality. Thus suggesting that a 1-point increase in serum albumin is associated with a 12.6% reduction in the hazard of death, whilst a 1-point increase in comorbidities is associated with 27.7% increase in the hazard of death, a 1-point increase in urea is associated with a 2.2% increase in the hazard of death and a 1-point increase in distress is associated with an 8.7% increase in the hazard of death. Therefore albumin, comorbidities, urea and distress were identified as potential covariates.

**Table 3:** Univariate survival analysis of demographic and clinical factors

Items	Hazard Ratio	***p*** value
Age	HR = 1.004 (0.981–1.029)	0.714
Gender	HR = 1.632 (0.897–2.970)	0.109
Education	HR = 0.604 (0.206–1.769)	0.358
eGFR	HR = 0.982 (0.962–1.001)	0.067
Time since diagnosis	HR = 0.042 (0.000–3.758)	0.167
Albumin	HR = 0.874 (0.822–0.928)	0.000
Haemoglobin	HR = 0.854 (0.717–1.018)	0.077
Comorbidities	HR = 1.277 (1.179–1.384)	0.000
Urea	HR = 1.022 (1.009–1.035)	0.001
Distress	HR = 1.087 (1.048–1.128)	0.000

The association between the eGFR level and mortality was non-significant (HR = 0.982, 95% CI: 0.962–1.001, *p* = 0.067). Whilst the time elapsed since diagnosis was not significantly associated with mortality in univariate associations. This might be attributable to the relatively small sample size. However, considering that all participants were exposed to a CKD diagnosis and that they all had varying degrees of eGFR, it was decided that these two factors would be also treated as covariates so as to ensure that these factors are accounted for due to their previous association with morbidity and mortality in CKD cohorts [1, 2].

Individual illness perception items were explored for their association with mortality in unadjusted and adjusted Cox proportional hazards. As outlined in model 1, Table 4; all the illness perceptions were found to be significantly associated with mortality. Adjusted models (model 2) controlled for albumin, comorbidities, urea, distress, eGFR levels and time elapsed. As outlined in Table 4; identity beliefs (HR = 1.199, 95% CI: 1.060–1.357, *p* = 0.004), perceptions of a chronic timeline (HR = 1.065, 95% CI: 1.003–1.132, *p* = 0.041), personal control beliefs (HR = 0.845, 95% CI: 0.748–0.955, *p* = 0.007) and perceptions of control over the treatment (HR = 0.812, 95% CI: 0.725–0.909, *p* = 0.000) remained significant predictors of mortality after controlling for all covariates.

**Table 4:** The association between illness perception items and survival

	Model 1	Model 2
Items	Unadjusted HR	Adjusted HR
**Identity**	1.239 (1.123–1.368)*	1.199 (1.060–1.357)*
**Timeline Acute / Chronic**	1.076 (1.014–1.142)*	1.065 (1.003–1.132)**
**Timeline Cyclical**	1.131 (1.046–1.221)*	1.029 (0.948–1.115)
**Consequences**	1.100 (1.039–1.164)*	1.052 (0.984–1.124)
**Personal Control**	0.786 (0.720–0.859)*	0.845 (0.748–0.955)*
**Treatment Control**	0.778 (0.713–0.849)*	0.812 (0.725–0.909)*
**Illness Coherence**	0.912 (0.841–0.990)**	0.929 (0.842–1.025)
**Emotional Representations**	1.114 (1.050–1.181)*	1.051 (0.966–1.144)

HR and 95% CI. * *p* < 0.01, **p < 0.05

Adjusted for serum albumin, comorbidity score, urea, distress, eGFR level and time elapsed

### Fully adjusted survival models

Finally a fully saturated model was loaded making use of Cox Regression analysis including the four illness perceptions items; identity, timeline, personal control and treatment control, as well as the covariates namely albumin, comorbidities, urea, distress scores, eGFR levels and time elapsed. in order to create a final model outlining the significant predictors of mortality in a CKD cohort.

Table 5 outlines the predictors of mortality and indicates that identity (HR = 1.215, 95% CI: 1.038–1.421, *p* = 0.015), treatment control (HR = 0.765 (0.635–0.922, *p* = 0.005) and chronic timeline (HR = 1.043 (1.008–1.080, *p* = 0.011) remained significant predictors of mortality together with serum albumin, comorbidities and urea. Thus suggesting that keeping all covariates constant; a 1- point increase in the perceived identity (i.e., the number of symptoms assigned) is associated with 21.5% increase in the hazard of death, a 1-point increase in perceived chronic timeline is associated with a 4.3% increase in the hazard of death, whilst a 1-point increase in treatment control is associated with a 23.5% reduction in the hazard of death. In this adjusted model, albumin, comorbidities and urea were also predictors of survival.

**Table 5:** Predictors of mortality in predialysis CKD (*n* = 200)

**Identity**	HR = 1.215 (1.038–1.421)	*p* = 0.015
**Treatment Control**	HR = 0.765 (0.635–0.922)	*p* = 0.005
**Timeline Acute/Chronic**	HR = 1.043 (1.008–1.080)	*p* = 0.011
**Albumin**	HR = 0.917 (0.848–0.992)	*p* = 0.031
**Comorbidities**	HR = 1.296 (1.155–1.454)	*p* = 0.000
**Urea**	HR = 1.027 (1.001–1.054)	*P* = 0.043

HR and 95% CI

Adjusted for personal control, distress score, eGFR level and time elapsed

## Discussion

The study aimed to assess the influence of illness perceptions on mortality in incident predialysis CKD patients. This study provides evidence that illness beliefs are associated with an increased risk of mortality in CKD patients, independent of other important clinical and demographic variables.

In univariate analyses, stronger perceptions of illness identity, timeline beliefs (both chronic and cyclical), greater perceived consequences and emotional representations were associated with increased mortality. In contrast, stronger beliefs regarding personal control, treatment control and illness coherence were associated with a reduction in mortality risk. It is speculated that these unhelpful illness beliefs are associated with poorer self-management behaviors which in turn may increase the risk of adverse outcomes. Evidence from other conditions has shown that illness perceptions predict self-management behaviours [30] and that psychological interventions designed to alter negative illness and treatment perceptions have successfully increased adherence to medication and physical activity [31]. Such interventional approaches are important to explore in CKD management programs to see whether they improve patient outcomes.

In adjusted analysis, patients who perceived more symptoms in relation to their condition (perceived identity) had an increased hazard of death. These findings coincide with those of Angels et al., (2015) [32] who found that as the CKD patients deteriorate, more symptoms are experienced and these are perceived by patients to be a consequence of kidney disease. In some cases patients report difficulty in attributing their physical symptoms to their CKD which may impact on the strength of their illness identity [33]. Misattributing symptoms of CKD and related treatment regimens could influence self-management behaviours such as medication adherence and physical activity. A study by Ricardo et al., (2013) [34] has reported that non-adherence to treatment regimen is associated with higher rates of all-cause mortality in chronic kidney disease and that strategies for improving self-management behaviours are vital for optimal clinical outcomes in the management of CKD. The fact that patients who reported more perceived symptoms suffered from higher mortality might also be associated with distress. A perception of increased severity on perceiving and interpreting symptoms has been associated with higher levels of distress amongst CKD patients [35]. Previous studies have linked depression to poor outcomes including inflammation, as well as non-adherence to therapy, an unhealthy lifestyle and poor nutrition [5, 6, 19].

Stronger chronicity timeline beliefs were also found to be associated with increased mortality in adjusted models. The presence of chronic symptoms, patient education and learned experiences from other patients contribute to this belief [36]. Difficultly in attributing symptoms to CKD may lead patients to perceive their illness as chronic, more unpredictable in nature, which could impact upon patients’ sense of control and adherence to self-management regimens.

Finally participants who perceived their treatment to be less effective (poor treatment control) had a higher hazard of mortality. This relationship remained stable after adjusting for clinical covariates. These results are consistent with previous studies [5, 16] in dialysis patients which also demonstrate an association between treatment control perceptions and mortality.

Taken together, the Common-Sense Self-Regulation Model (CS-SRM) provides explanation in terms of that beliefs about the treatment or medication being ineffective could influence the extent to which patients feel motivated to regulate their illness and to adhere to treatment guidelines, which may seriously increase their mortality risk [12, 13].

In conclusion, CKD patients’ perceptions of treatment control, perceptions of a chronic timeline and perceived illness identity predict survival independently of comorbidities, serum albumin and urea. Further studies are required to replicate these findings and to provide evidence for the suggested mechanism.

### Impact of the study

To our knowledge, this is the first report showing that patients’ illness perceptions regarding their identity, timeline and treatment control are related to survival in predialysis CKD patients. The present study shed more light on the predictors of mortality in predialysis CKD and yielded considerable evidence for the positive relationship between illness perceptions and mortality in CKD. It has confirmed that the illness identity, a chronic timeline and poor treatment control were significant predictors of mortality in pre-dialysis CKD.

These findings provide additional support for the role of cognitive and emotional representations, and the interplay with compliance to treatment and disease prognosis in CKD [12, 13]. In addition, if perceptions regarding identity and treatment control indeed pose a serious risk factor for mortality, there is hope since as evidenced by previous studies, in contrast to other risk factors such as age and comorbidity, illness perceptions prove to be modifiable by psycho-educational interventions [20, 37]. Thus, according to the results, by focussing on changing illness perceptions as a treatment goal, may improve both the psychological adjustment and regulation and as well as disease prognosis in CKD.

### Limitations

This study has several strengths; a good sample of predialysis CKD participants and a good consent rate, a good response rate to questionnaires, since questionnaires were completed in the presence of the lead researcher and good matching of data, with clinical markers being collected in line with the psychological markers in terms of timeframe, so as to ensure congruence between the different factors. However, the study had also a number of weaknesses, whilst being one of the first studies looking at illness perceptions and mortality in early stage predialysis CKD patients; the sample was all recruited from the local nephrology outpatient clinics, thus capturing the service users that make use of government services only. In addition, the study looks at all-cause mortality as the outcome measure and doesn’t discriminate against the different types of deaths thus preventing, in this way, the possibility to conduct discriminatory analysis to test for any associations between illness perceptions and different causes of death. Thus, our results need to be interpreted with some caution.

## Conclusions

In conclusion, our findings have demonstrated that illness perceptions are associated with mortality in CKD. In our study, negative beliefs regarding the severity of CKD, the timeline and poor treatment control predicted increased risk of all-cause mortality, independent of other known clinical covariates associated with disease severity. Interestingly distress failed to predict mortality in our cohort. Further studies are required to test whether the assessment and the implementation of psychological interventions aimed at modifying maladaptive illness perceptions influence clinical outcomes in CKD.

## Data Availability

The data sets used and analysed during the current study are available from the corresponding author on reasonable request.

## Abbreviations

CKD: Chronic Kidney Disease
ESRD: End Stage Renal Disease
CS-SRM: Common-Sense Self-Regulation Model
MMSE: Mini Mental State Examination
eGFR: estimated Glomerular Filtration Rate
CRP: C Reactive Protein
IPQ–R: Revised Illness Perception Questionnaire
IP: Illness Perceptions
PHQ-9: Patient Health Questionnaire
GAD-7: Generalized Anxiety Disorder 7-item scale
PHQADS: Patient Health Questionnaire and Generalized Anxiety Disorder
